# Detection of human adenoviruses in influenza-negative patients with respiratory tract infections in Nanning, China

**DOI:** 10.1186/s12985-023-02093-0

**Published:** 2023-08-02

**Authors:** Jianqiu Qin, Tengyue Yan, Liujiang Yin, Cheng Yang, Liang Wang, Hong Qiu, Yanling Hu, Bin Xu

**Affiliations:** 1 Nanning Center for Disease Control and Prevention, Nanning, Guangxi, 530023 China; 2 Collaborative Innovation Centre of Regenerative Medicine and Medical Bioresource Development and Application Co-constructed by the Province and Ministry, Guangxi Medical University, Nanning, China; 3 Institute of Life Sciences, Guangxi Medical University, Nanning, Guangxi, 530021 China

**Keywords:** Human adenoviruses, Respiratory infections, Nanning, Prevalence, Hexon gene

## Abstract

**Background:**

Human adenoviruses (HAdV) have been known to cause a range of diseases, including respiratory tract infections (RTIs). However, there is limited information available regarding the genotype diversity and epidemiology of HAdV associated with RTIs in Nanning.

**Methods:**

Between June 2019 and December 2021, throat swab, nasal swab, or nasopharyngeal swab samples were obtained from individuals hospitalized with respiratory tract infections (RTIs). Statistical software was used to analyze the epidemiological data. The highly conserved 132-bp gene region of the HAdV hexon was targeted for the detection of HAdV using a qPCR assay. An 875-bp hexon gene fragment was subjected to phylogenetic analysis.

**Results:**

Significant variations were observed in the age and gender distribution of HAdV-positive patients (P = 0.004 and P = 0.025, respectively). The age distribution of HAdV-positive patients showed that 67.89% of those who tested positive were the age group of 0–6 years. Furthermore, the prevalence of HAdV detection was highest during spring and autumn, with a peak in February. Additionally, genotyping of the 36 HAdV-positive samples with 875-bp fragments identified the presence of circulating HAdV species B, C, and E in Nanning between 2019 and 2021.

**Conclusions:**

This study identified an association between HAdV prevalence and age as well as season. Among hospitalized patients with RTIs in Nanning, HAdV-B, HAdV-C, and HAdV-E were found to be co-circulating. The most commonly detected genotypes were HAdV-C1, HAdV-C6, and HAdV-E4.

**Supplementary Information:**

The online version contains supplementary material available at 10.1186/s12985-023-02093-0.

## Background

Human adenoviruses (HAdV) are DNA viruses that belong to the Adenoviridae family and lack an enveloped structure. Their genome is approximately 36Kb in size and encodes about 40 proteins, including Penton base, Hexon, and Fiber proteins [1]. HAdV can infect various tissues and organs, causing a range of illnesses with varying severity, such as sore throat, rhinitis, fever, pneumonia, and bronchitis [2, 3]. While anyone can be affected by HAdV, individuals with weakened immune systems, cardiovascular disease, and young children are at a higher risk of developing severe forms of the disease [4, 5].

HAdV infections are often underreported worldwide, and the distribution of adenovirus types can vary across different geographic regions and human populations. To date, approximately 110 HAdV genotypes have been identified and classified into seven species (A-G) within the Mastadenovirus genus based on their physical, chemical, and biological properties [6]. Among these, genotypes 3, 4, 7, 14, 21, and 55 are known to cause severe infections and have been linked to global outbreaks [7–9]. In recent years, new HAdV genotypes with novel pathogenicity and epidemic characteristics have emerged, posing new threats and challenges to public health [10]. Therefore, it is essential to investigate the molecular epidemiological characteristics of HAdV-induced acute respiratory tract infections.

HAdV particles are composed of 252 putamens, including 240 hexons, 12 pentons, and 12 fibers. Hexons play a critical role as type-specific and species-specific antigens that are highly susceptible to immune selection. The hypervariable regions (HVR1-7) within hexons contain numerous antigen epitopes, making them particularly sensitive to immune selection. A recent study conducted in Nanning, China, from June 2019 to December 2021 aimed to investigate the epidemiological, clinical, and molecular characteristics of HAdV infections among patients with respiratory tract infections (RTIs) who tested negative for influenza. The study also explored the link between HAdV infection and RTI symptoms to provide insights into controlling and preventing HAdV infection in China.

## Materials and methods

### Patient specimens

In this study, a total of 8,315 samples were collected from patients with respiratory tract infections (RTIs) at Nanning First People’s Hospital and Nanning Maternal and Child Health Care Hospital in Nanning, China, between June 2019 and December 2021. All participants were fully informed about this influenza epidemiological surveillance and provided written informed consent prior to enrollment in the study enrollment in the study. An average of 280 samples were collected per month during the study. RTI illness was defined as the presence of at least two of the following influenza-like symptoms within three days: fever, cough, nasal obstruction, expectoration, sneezing, and dyspnea. The collected specimens were stored in Yocon virus preservation solution (MT0301), which contained Hank’s medium with streptomycin, antifungal antibiotics, BSA (V), cryoprotectant, biological buffer, and amino acids. The samples were transported to the laboratory on ice and stored at -80℃ until further processing. Clinical data of the patients were obtained from the hospital database.

### Screening for influenza viruses in initial RTI patients

To exclude RTI patients who tested positive for influenza, all RTI patients underwent initial screening. Total viral nucleic acids were extracted from 200 µL of each clinical specimen using the Prefill Viral Total NA Kit 4 × 48 preps for KingFisher FLEX (Fisher Scientific LabServ), following the manufacturer’s instructions. The detection of influenza viruses was carried out using a qPCR assay that targeted specific primers for A type (Forward: 5’-GAC CRA TCC TGT CAC CTC TGA C-3’; Reverse: 5’-GGG CAT TYT GGA CAA AKC GTC TAC G-3’) and B type (Forward: 5’-TCC TCA ACT CAC TCT TCG AGC G-3’; Reverse: 5’-CGG TGC TCT TGA CCA AAT TGG-3’), with a common probe for A and B types (5’HEX-CCA ATT CGA GCA GCT GAA ACT GCG GTG-BHQ2 3’). The qPCR cycling program included 50 °C for 30 min, 95 °C for 15 min, 5 cycles of 95 °C for 15 s, and 50 °C for 30 s, 72 °C for 1 min, followed by 40 cycles of 95 °C for 15 s, and 55 °C for 45 s. Samples with a cycle threshold (Ct) value < 38 were considered positive for influenza virus.

### The inspection method of HAdV in influenza-negative specimens

The total nucleic acid of each clinical specimen was extracted by using the Prefill Viral Total NA Kit 4 × 48 preps for KingFisher FLEX(Fisher Scientific LabServ)according to the manufacturer’s instructions. HAdV detection was performed using qPCR assay targeting the highly conserved gene region (132-bp) of the HAdV hexon. QuantiTect Probe RT-PCR Kit (200)(QIAGEN) (AppliedBiosystems, USA) was used to amplify HAdV hexon DNA using specific primers (Forward:5’-CAGGACGCCTCGGRGTAYCTSAG-3’; Reverse: 5’-GGAGCCACVGTGGGRTT-3’) and probe (5’FAM-CCGGGTCTGGTGCAGTTTGCCCGC-BHQ1-3’). The qPCR cycling program included 50 °C for 30 min, 95 °C for 15 min, followed by 5 cycles of 95 °C for 15 s, 50 °C for 30 min, and 72 °C for 1 min, follow by 40 cycles of 95 °C for 15 s, and 60 °C for 45s. Samples with acycle threshold (Ct) < 38 were regarded as positive.

### HAdV genotyping and phylogenetic analysis

HAdV genotyping was performed using nested PCR targeting the hypervariable region [11]. The primers used were forward 5’-TTCCCCATGGCNCACAACAC-3’ and reverse 5’-GCCTCGATGACGCCGCGGTG-3’. Samples that failed to amplify were defined as untyped. The nested-PCR products were sequenced by TaKaRa LA Taq with GC Buffer(Takara Code No.RR02) and the acquired hexon gene sequences were used together with 26 HAdV strain sequences downloaded from GenBank to construct the phylogenetic tree. The Maximum Likelihood (ML) method in MEGA 7.0 software was used for phylogenetic analysis. The evolutionary tree was evaluated using 1000 bootstrap replicates to verify the HAdV genotypes.

### Statistical analysis

The percentage of HAdV-positive specimens was calculated by dividing the number of positive samples by the total samples collected during the same time period. The detection rates of HAdV among different populations and seasons were evaluated using the Chi-square and Fisher’s exact tests, with P-values < 0.05 considered statistically significant. Sequence alignment and phylogenetic analysis were conducted using MEGA7.0. Epidemiological data analysis and graph drawing were performed using SPSS26.0 and R 4.2.0 software.

## Results

### Characteristics of patients

During the period from June 2019 to December 2021, a total of 2838 samples were collected from Nanning First People’s Hospital,1405 (49.51%) were male and 1433 (50.49%) were female, resulting in a sex ratio of 0.98:1 (Additional file 1: Table S1). A total of 5477 samples were collected from Nanning Maternal and Child Health Care Hospital,3240 (59.16%) were male and 2237 (40.84%) were female, resulting in a sex ratio of 1.45:1 (Additional file 1: Table S2). A total of 8315 samples were collected from the two hospitals; among them, 4645 (55.86%) were males, and 3670 (44.14%) were females, resulting in a sex ratio of 1.27:1. The age range was from 1 month old to 95 years old with an average age of 12 years and a median age of 4 years old (IQR: 1–20 years old). HAdV is prone to infect immunocompromised people, especially in young children who lack humoral immunity [3, 12, 13]. For further comparison, patients were categorized into five age groups: infants (0 ~ 6 year), youth (~ 20 year), prime of life (~ 40 year), middle age (~ 60 year) and the elderly (~ 95year). Out of all patients, 218 (2.62%) tested positive for HAdV infection, and there was a significantly different detection rate among age groups (P = 0.004). Among the 218 HAdV-positive patients, 138 (63.30%) were male, and 80 (36.70%) were female, with a sex ratio of 1.725:1 (Table 1). Additionally, we randomly selected 100 influenza positive patients to dest HAdV, and found 3.39% HAdV-positive patients.

**Table 1:** Age and sex differences among hospitalized patients with HAdV infection

Variable	Number of patient	Number of patient positive for HAdV	Percentage patient positive for HAdV (%)	*P* value
Age (years)				
0 ~ 6	4888	148	3.44	0.004
~ 20	1385	40	2.89	
~ 40	1512	22	1.46	
~ 60	324	7	2.23	
~ 95	206	1	0.49	
Gender				
Male	4645	138	2.97	0.025
Female	3670	80	2.18	
Total	8315	218	2.62	

### Epidemiology of HAdV

**Fig. 1:**
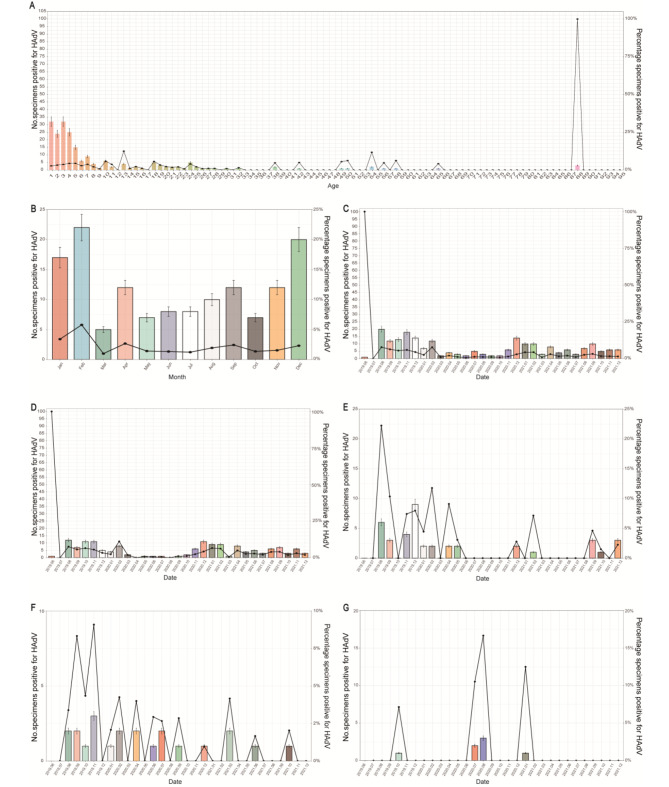
The distribution of HAdV infections across age groups over different time periods. **(A)** HAdV Infection Distribution across Age Groups (0–95 years). **(B)** Monthly HAdV infection status from 2020–2021. **(C)** HAdV infection in each month from June 2019 to December 2021. **(D)** Monthly HAdV infection status in the 0–6 years old group from June 2019 to December 2021. **(E)** Monthly HAdV infection status in the 7–20 years old group from June 2019 to December 2021. **(F)** Monthly HAdV infection status in the 21–40 years old group from June 2019 to December 2021. **(G)** Monthly HAdV infection status in the 41–60 years old group from June 2019 to December 2021

The 0–6 age group had the highest rates of HAdV infection, followed by the 7–20 age group, then the 21–40 age group. The lowest rates were observed in the 71–95 age group (Fig. 1A). There was a significant variation in the prevalence of HAdV infection throughout the years 2020 and 2021 (P < 0.001), with the highest detection rate occurring from August to February (71.43%, 100 out of 140 cases), and a peak in February (15.71%, 22 out of 140 cases) (Fig. 1B). The infection of HAdV in different periods is shown in Fig. 1C. The prevalence of HAdV infection across all four age groups mirrored the overall prevalence over the months from June 2019 to December 2021 (Fig. 1D-G). These findings suggest that HAdV infection is seasonal and may be influenced by age.

### HAdV genotyping and phylogenetic analysis

**Fig. 2:**
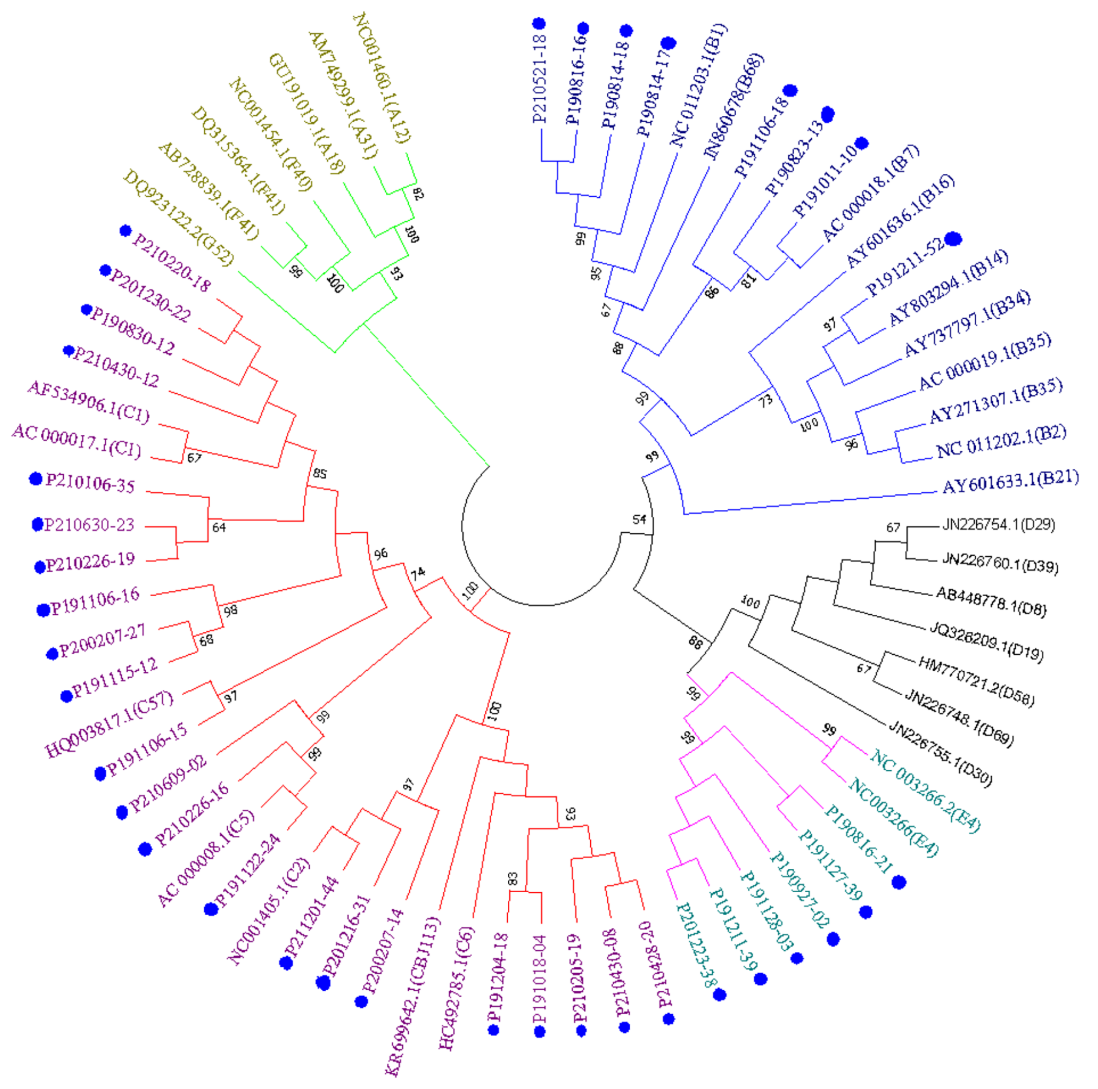
Phylogenetic analysis of HAdV hexon gene (758-bp) compared with reference strains. The strains that sequencing by our primers is marked with “●”

After amplifying the hexon gene using nested-PCR products with Sanger sequencing, 36 HAdV-positive samples with 875-bp fragments were obtained and successfully genotyped. In our research project, we conducted an analysis of 36 samples using phylogenetic methods with maximum likelihood method in MEGA 7.0 based on the hexon gene sequence. Our findings indicate the presence of three distinct HAdV genotypes. The phylogenetic analyses showed that 8 cases belonged to species B (HAdV-B1, HAdV-B7, HAdV-B14), 22 belonged to species C (HAdV-C1, HAdV-C2, HAdV-C5, HAdV-C57, HAdV-C6), and 6 belonged to species E (HAdV-E4). HAdV-C1 was the most prevalent genotype (10/36), followed by HAdV-C6 and HAdV-E4 (Fig. 2). These results indicate that species B, species C and species E was circulated in Nanning from 2019 to 2021, making up 22.22%, 61.11% and 16.67% of the isolates, respectively.

## Discussion

HAdV is associated with various clinical manifestations, including hepatitis,gastroenteritis, meningitis, cystitis, conjunctivitis, upper and lower respiratory tract infections, and myocarditis etc. [14–20]. Moreover, HAdV infection spreads easily, is highly contagious in some cases, can cause local outbreaks, is associated with a severe course, and occasionally leads to fatal outcomes even in immunocompetent individuals [21–25]. Most HAdV species are observed to have a global circulation, but the predominant types vary among countries or geographic regions and undergo changes over time [26–28]. An increased number of HAdV types are now emerging from genomic analyses. Most importantly, there is no standardized clinical treatment, HAdV is also difficult to prevent,and the ECIL does not recommend prophylactic antiviral therapy with currently available virustatic drugs [29]. Therefore, our objective was to ascertain the distribution of HAdV infections by age and gender in these hospitals and to monitor the prevalence of HAV. Additionally, we examined the genetic diversity of HAV among different age groups and months.

Human adenovirus (HAdV) can cause respiratory tract infections (RTIs) with clinical symptoms that resemble those caused by other respiratory pathogens such as influenza and parainfluenza viruses [30]. This makes it challenging to diagnose HAdV infections based solely on symptoms. Therefore, rapid and effective diagnostic methods are crucial for identifying and genotyping HAdV. In this study, a qPCR assay was developed to detect and quantify HAdV in nasal and pharyngeal aspirate (NPA) specimens collected from 8,315 individuals. Out of the total specimens screened, 218 (2.62%) tested positive for HAdV, which is lower than the reported prevalence of HAdV in conjunction with avian influenza (ranging from 1.70 to 13.90%) [11, 31, 32]. In addition, the detection rate of HAdV varied across different regions in China. For instance, in Zhejiang province from 2006 to 2012, the detection rate of HAdV in hospitalized children with acute lower respiratory tract infections was 0.63%, while in Shenzhen city from 2012 to 2015, it was 2.24% [33, 34]. This study also found differences in the detection rate based on sex composition, age distribution, and seasonal effects.

Our study found that the highest prevalence of HAdV occurred during the autumn and winter seasons (71.43% from August to February), and the peak prevalence was observed in February. Similar seasonal patterns have been observed in other studies conducted in Northern China and Mexico [35, 36]. However, some previous studies have suggested that the detection rates of HAdV are positively associated with the monthly mean temperature and sunshine duration, while being negatively correlated with wind speed [7]. It should be noted that Nanning experiences a prolonged period of high temperatures from May to October, with the average daily maximum temperature exceeding 30 °C. From November to April, the average temperature is around 15 °C. This may account for differences in the results of this study compared to those conducted in other cities.

This study indicates that HAdV infections are predominantly observed in children under the age of 6, accounting for 67.89% of cases, highlighting the importance of HAdV as a pediatric pathogen. These findings are consistent with previous studies that have also reported HAdV infections to be common in young children [32, 37, 38]. HAdV can be easily transmitted through fomites contaminated with infectious bodily fluids. Interestingly, our study found that the detection rate of HAdV was lowest among individuals over the age of 60 (0.45%). However, these findings need to be interpreted with caution as the sample size was small, and longer observation periods may be required to confirm the results.

Our study employed nested-PCR to genotype 218 HAdV samples, resulting in 36 hexon 875-bp sequence. Phylogenetic analysis of these sequences revealed that 8 cases belonged to species B (B1, B7 and B14), 22 cases belonged to species C (C1, C2, C5, C6, C57), and 6 cases belonged to species E(E4). The most commonly found genotype was HAdV-C1 (10/36), followed by HAdV-C6 and HAdV-E4. Several studies have previously reported that the most common HAdV species causing respiratory tract infections (RTIs) in children worldwide are B (B3, B7, B21), C (C1, C2, C5, C6), and E (E4) [39–41]. In China, HAdV species B, C, and E are associated with severe pneumonia and are also the most prevalent species. In this study, phylogenetic analysis of 36 samples based on the hexon gene sequence revealed three HAdV genotypes. The results showed that HAdV species B and C were the most commonly found, accounting for 22.22% and 61.11% of isolates, respectively.

## Conclusions

This three-year study investigated the prevalence, age distribution, seasonality, and molecular epidemiology of human adenovirus (HAdV) infections associated with respiratory tract infections (RTIs) in Nanning, China, excluding avian influenza. The findings suggest recent changes in the circulating HAdV genotypes associated with RTIs in Nanning. These changes could aid in improving the diagnosis of HAdV-related diseases and developing new detection, treatment, and prevention strategies for clinical settings. However, only 36 sequences of the hexon gene were amplified from 218 HAdV samples in this study. Therefore, further analysis of the full-length genome is necessary to understand the extent of genetic variation and any potential recombination that occurred in the strains identified in this study.

## Electronic supplementary material

Below is the link to the electronic supplementary material.

Supplementary Material 1

Supplementary Material 2

## Data Availability

Condensed anonymized data are available from the corresponding author on reasonable request.

## Abbreviations

Ct: cycle threshold
HAdV: Human adenoviruses
ML: Maximum Likelihood
MEGA: Molecular evolutionary genetics analysis
NPA: nasal and pharyngeal aspirate
RTIs: respiratory tract infections
qPCR: quantitative real-time polymerase chain reaction
SPSS: Statistical product and service solutions
