# Supplementary material for: Detection of human adenoviruses in influenza-negative patients with respiratory tract infections in Nanning, China
Source: Virol J. 2023 Aug 2;20:171. doi: 10.1186/s12985-023-02093-0 (PMC10398977; doi:10.1186/s12985-023-02093-0)
Supplement: Supplementary file 1 — Supplementary Material 1 [file 12985_2023_2093_MOESM1_ESM.docx]

Table S1 Age and sex differences among Nanning First People's Hospital patients with HAdV infection

| **Variable** | **Number**  **of**  **patient** | **Number of**  **patient positive**  **for HAdV** | **Percentage**  **patient positive**  **for HAdV (%)** | ***P* value** |
| --- | --- | --- | --- | --- |
| Age (years) |  |  |  |  |
| 0 ~ 6 | 124 | 14 | 11.29 | <0.001 |
| ~ 20 | 679 | 25 | 3.68 |  |
| ~ 40 | 1505 | 22 | 1.46 |  |
| ~ 60 | 324 | 7 | 2.16 |  |
| ~ 95 | 206 | 1 | 0.49 |  |
| Gender |  |  |  |  |
| Male | 1405 | 39 | 2.78 | 0.238 |
| Female | 1433 | 30 | 2.09 |  |
| Total | 2838 | 69 | 2.43 |  |
