# Supplementary material for: Detection of human adenoviruses in influenza-negative patients with respiratory tract infections in Nanning, China
Source: Virol J. 2023 Aug 2;20:171. doi: 10.1186/s12985-023-02093-0 (PMC10398977; doi:10.1186/s12985-023-02093-0)
Supplement: Supplementary file 2 — Supplementary Material 2 [file 12985_2023_2093_MOESM2_ESM.docx]

Table S2 Age and sex differences among Nanning Maternal and Child Health Care Hospital patients with HAdV infection

| **Variable** | **Number**  **of**  **patient** | **Number of**  **patient positive**  **for HAdV** | **Percentage**  **patient positive**  **for HAdV (%)** | ***P* value** |
| --- | --- | --- | --- | --- |
| Age (years) |  |  |  |  |
| 0 ~ 6 | 4764 | 134 | 2.81 | 0.523 |
| ~ 20 | 706 | 15 | 2.12 |  |
| ~ 40 | 7 | 0 | 0 |  |
| ~ 60 | 0 | 0 | - |  |
| ~ 95 | 0 | 0 | - |  |
| Gender |  |  | - |  |
| Male | 3240 | 99 | 3.06 | 0.067 |
| Female | 2237 | 50 | 2.24 |  |
| Total | 5477 | 149 | 2.72 |  |
